# Premature Calcifications of Costal Cartilages: A New Perspective

**DOI:** 10.1155/2014/523405

**Published:** 2014-12-23

**Authors:** Walter Rhomberg, Antonius Schuster

**Affiliations:** ^1^Department of Radiooncology, General Hospital, Carinagasse 47, 6800 Feldkirch, Austria; ^2^Department of Radiology, General Hospital, 6900 Bregenz, Austria

## Abstract

*Background*. Calcifications of the costal cartilages occur, as a rule, not until the age of 30 years. The knowledge of the clinical significance of early and extensive calcifications is still incomplete. *Materials and Methods*. A search was made to find patients below the age of 30 years who showed distinct calcifications of their lower costal cartilages by viewing 360 random samples of intravenous pyelograms and abdominal plain films. The histories, and clinical and laboratory findings of these patients were analyzed. *Results*. Nineteen patients fulfilled the criteria of premature calcifications of costal cartilages (CCCs). The patients had in common that they were frequently referred to a hospital and were treated by several medical disciplines. Nevertheless many complaints of the patients remained unsolved. Premature CCCs were often associated with rare endocrine disorders, inborn errors of metabolism, and abnormal hematologic findings. Among the metabolic disorders there were 2 proven porphyrias and 7 patients with a suspected porphyria but with inconclusive laboratory findings. *Conclusion*. Premature CCCs are unlikely to be a normal variant in skeletal radiology. The findings in this small group of patients call for more intensive studies, especially in regard to the putative role of a porphyria.

## 1. Introduction

The calcification of the costal cartilages follows gender-related patterns and is generally not evident radiographically until after the age of 30 years [1]. The forms and the onset of these calcifications were used to determine the gender and the age of unknown bodies in the forensic medicine [2–4]; however, the clinical significance of premature calcifications of the lower rib cartilages is not fully elucidated as yet. In case reports, there have been descriptions of an association of premature calcifications with hyperthyroidism [5, 6], corticosteroid medication [7], and rare congenital diseases such as adrenogenital syndrome [8, 9] or Keutel syndrome [10, 11], but some authors regard even extensive costal cartilage calcifications (CCC) as a normal finding or variant [12].

Some forty years ago, around 1968, a search was made for premature CCCs according to the criteria described below. Primary data have originated from the Kantonsspital Winterthur in Switzerland at the suggestion of the late Dr. Walter Bessler. We collected the clinical data of 14 patients with premature CCCs, but we felt at that time the data were somewhat inconclusive, and so we did not submit them for publication. However, we found the data interesting and kept them for almost three decades. A renewed interest was triggered by the detection of a case of porphyria associated with glioblastoma multiforme [13] due to the finding of extensive CCC and our recall of suspected porphyrias in relation to CCC found in that earlier investigation. Now, few further cases of CCC were analysed and added to the old cases. They continue to pose interesting and unsolved questions. It has to be mentioned that at the time of 1968 no ethical committees were known in the frame of radiodiagnostic research.

The aim of this paper is to call for further research in this area since it is unlikely that premature calcifications of costal cartilages are normal variants in skeletal radiology.

## 2. Materials and Methods

**Figure 1:**
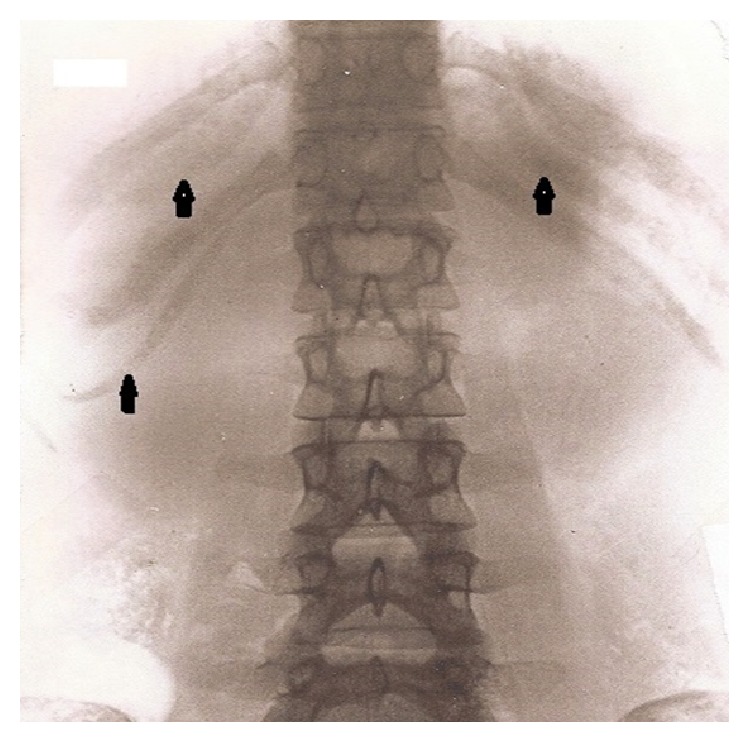
Almost complete calcification of the costal cartilages in a 20-year-old woman. See arrows.

A search was made for patients who showed extensive or complete calcifications of their lower costal cartilages and whose age was below 30 years. Premature costal cartilage calcifications (CCCs) were found by viewing intravenous pyelograms and plain radiographs of the abdomen. In these images the calcifications are clearly visible and might be quantified more precisely compared to CT images (Figure 1). In case of abnormal calcifications, permission was obtained to study the charts of those patients that were treated in different departments of the hospital. The histories, the clinical and laboratory findings, and the suspected diagnoses of 19 such patients were analysed and put together. Thereby, it soon became evident that it would hardly be possible to present complete and conclusive scientific data for the simple reason that we as radiologists had little influence to urge all the necessary work-up that had to be done in several patients with CCCs.

In order to roughly assess the frequency of CCC, we have screened about 360 abdominal plain films and intravenous pyelograms of people in the age of 18 to 30 years by taking random samples. Nineteen cases of pronounced CCC were found, corresponding to a rate of 5.3% related to these specific radiographic views.

Calcifications were termed complete if the whole cartilage from the costal to the sternal end was calcified area-wide. Intensive or advanced cartilage calcification was noted if at least 50% of the area was involved, independent of the form of calcifications which might be different in males and females. Some patients showed only linear calcifications at the costochondral border; they were classified as negative.

## 3. Results

By spot checks, pronounced CCCs were diagnosed in 19 young patients. There were 17 females and 2 males. The median age was 27 years (range, 19–31).

### 3.1. General Clinical Observations

The most frequent reason for a hospitalization was abdominal pain of unknown origin in 14 of 19 cases. The patients had in common that all but one had to undergo a work-up in several medical disciplines, most often in the departments of internal medicine, gynecology, surgery, neurology, casualty ward, and urology. Up to 29 hospital referrals within 9 years were noted in one patient. In the history of the patients a proneness to infections was observed. In 13 cases disturbances at the urogenital system, mainly pyelonephritis or cystitis, were diagnosed. Many symptoms remained unclear, and abdominal pain attacks recurred over years.

### 3.2. Metabolic and Endocrine Disorders

Seven patients had clinical symptoms suggestive of a porphyria, but the available laboratory tests were not unequivocal or even not done. Three patients showed abnormal levels of delta-aminolevulinic acid (ALA) and porphobilinogen in the urine, but only two patients were ultimately proven cases of an unspecified porphyria. The majority of the patients had metabolic disturbances, and some of them showed rare diseases (Table 1).

Noticeable are pathological findings of endocrine organs. Addison's disease was diagnosed twice and hyperthyreosis in one case, and degenerative or neoplastic diseases of the gonads are diagnosed in 5 cases. The menstrual cycle was disturbed in further 5 patients. One case of Addison's disease was unusual in so far as the case presented as an idiopathic, familial form that showed normal levels of sodium and potassium in the serum. The disease was tolerated without hormonal substitution from the 8th to the 30th year of his life. The main respective findings of the patients were summarized in Table 1.

### 3.3. Hematologic Findings

A variety of hematologic alterations were noted. Eight patients (42%) presented with anemia of moderate to severe degree. Among them, the rare combination of thalassemia minor and intermittent polyglobulia was found in patient #9, and a cyclic pancytopenia occurred in patient #12. In patient #11, an inherited Morbus Addison was associated with an idiopathic thrombopenia between 40 and 70 G/l combined with a cyclic leukopenia. The bone marrow of the last two patients showed a severe disorder of the maturation of the erythropoiesis and thrombo- and myelopoiesis. Three other patients showed a strong shift to the left in the differential leukocyte counts over months, for example, frequency of bands 30–40%, normal values 4–15%. There was no clear reason for this shift to the left.

## 4. Discussion

Disorders of endocrine organs were found in an unusual frequency in our patients. Already in 1955, Fischer suggested to search for endocrine disorders in case of premature calcifications of the rib cartilages [14]. Among later reports of diseases associated with premature CCC, there were indeed observations of hyperthyroidism [5, 6] or of cases with adrenogenital syndrome [8, 9]. The full picture of an adrenogenital syndrome could never be proven in our patients, but some of its symptoms were seen, for example, a partial insufficiency of the suprarenal glands or an increased excretion of androgen metabolites in the urine. Interestingly, in Addisons's disease extensive calcifications of the ear cartilages were described [15]. However, it seems to be of limited value to deal here with the details of the extensive experimental work concerning the influence of hormones on CCC which was performed in earlier decades [16–19]. 

Main complaints were related to recurrent abdominal pain. Somehow, this may be due to the selection of intravenous pyelography and abdominal plain films for screening of CCC, and this of course implies a prevalence of urogenital and abdominal disorders to some degree. On the other hand, the origin of the abdominal pain attacks remained often unexplained in this cohort of patients. In the context of an abnormal excretion of porphobilinogen in the urine of three patients, one has to consider the occurrence of true porphyrias. At present, there is lack of certainty on the diagnosis of acute intermittent porphyria in some cases since the adequate work-up was not done and since there is obviously a lack of being familiar with these diseases.

During the last two decades important advances in the understanding of the porphyrias were made: specific enzyme deficiencies have been demonstrated, and genes have been isolated and located [20]. Thereby it became evident that there is a great deal of genetic heterogeneity in each porphyria [21]. Schattenberg and De Pauw suggested that, for instance, acute intermittent porphyria is not a very rare disease. Its manifestations vary and may lead to different clinical pictures [22]. This might be a reason why the abnormal porphyrin levels in the urine could not with certainty be linked to the diagnosis of a known form of porphyria in some of our cases.

There were several hematologic abnormalities among our patients, sometimes associated with a cyclic course. It remains open whether these abnormalities are independent diseases or are related to variants of a porphyria. Hematopoietic alterations within porphyric disorders are not well defined. Anemias may occur in some patients. Oubre et al. [23] described an association of porphyria cutanea tarda with a myelodysplastic syndrome, and in the literature there are some other case reports on the association of hematologic malignancies and porphyria.

Taken together, premature CCC seems to be associated with a variety of endocrine and/or metabolic disorders, acquired or inherited. It is possibly a mine for the diagnosis of rare diseases, and we think that the radiologic sign of premature CCC is always a pathologic finding; that is, premature CCCs are hardly a normal variant in skeletal radiology. Apart from Keutel syndrome [24], the common pathway leading to premature calcifications is unknown as yet. The part played by and the ratio of potential porphyrias in CCC remain likewise unclear. The conditions that may lead to premature CCC and the possible pathogenesis of premature CCCs are summarized in Table 2. The frequent occurrence of unclear abdominal pain syndromes in this small cohort of patients calls for further investigations by colleagues from internal medicine or specialists of porphyrias. If an association of premature CCC and porphyrias could be confirmed, this simple sign in abdominal X-rays would facilitate the thinking about the diagnosis of a porphyria which is still overlooked too often. So, in the absence of conclusive scientific clinical data, the relevance of this work is to induce further studies as to the interesting phenomenon of CCCs.

## Figures and Tables

**Table 1:** Selected clinical findings in 19 patients with premature calcifications of the costal cartilages.

Number	Age/Sex	Metabolic disorders	Diseases of endocrine organs	Laboratory data (increases)
1	30/M		Testicular fibrosis, azoospermia	
2	19/F	Acute intermittent porphyria		Delta-ALS + porphobilinogen
3	22/F		Mayer-von Rokitansky-Küster syndrome	
4	20/F	Suspected porphyria	Ovarian teratomas, bilateral	∗
5	25/F	Glucosuria, cholelithiasis		
6	26/F		Morbus Addison	
7	30/F	None	None	
8	20/F		Hyperthyroidism, goiter oligomenorrhea	
9	31/F	Suspected porphyria		17-OH-steroids^*^
10	27/F	Suspected porphyria	Microcystic degeneration of the right ovary	Porphobilinogen
11	30/M		Morbus Addison, inherited	
12	28/F	Decreased 17-keto- and 17-OH-steroids	Bilateral ovarian cysts	Delta-ALS + coproporphyrin
13	23/F	Orotic aciduria, suspected porphyria		∗
14	27/F	Cholelithiasis		
15	30/F	Suspected porphyria		∗
16	26/F	Cholelithiasis		
17	29/F	Suspected porphyria	Oligomenorrhea	∗
18	30/F	Suspected porphyria	Ovarian cysts, follicular	∗
19	27/F	Porphyria, unspecified	Hypothyroidism	

ALS: delta-aminolevulinic acid; ^*^no lab screening as to porphyria done.

**Table 2:** Conditions that may lead to premature calcifications of costal cartilages.

Clinical disorder	Presumed pathogenesis	References
Adolescent hyperthyroidism	Advanced bone maturation by toxic hormone doses	[1, 5, 6]

Exposure to corticoids	Complex findings in vitro; extensive research in earlier decades; direct influence on cartilage matrix	[7, 16, 17, 19]

Adrenogenital syndrome	Unknown	[8, 9]

Keutel syndrome	Autosomal recessive disorder with several anomalies; frequent consanguinity, description, 1971	[10, 11]
	Mutations in the matrix Gla protein gene (MGP) that acts as a calcification inhibitor (for details see [24])	[24]

Porphyria	Unknown	This paper

Systemic conditions as chronic renal failure or autoimmune disorders	Unknown	[1]
